# Clinically Relevant Reactivation of Polyomavirus BK (BKPyV) in HLA-A02-Positive Renal Transplant Recipients Is Associated with Impaired Effector-Memory Differentiation of BKPyV-Specific CD8^+^ T Cells

**DOI:** 10.1371/journal.ppat.1005903

**Published:** 2016-10-10

**Authors:** Michiel C. van Aalderen, Ester B. M. Remmerswaal, Kirstin M. Heutinck, Anja ten Brinke, Mariet C. W. Feltkamp, Neelke C. van der Weerd, Karlijn A. M. I. van der Pant, Frederike J. Bemelman, René A. W. van Lier, Ineke J. M. ten Berge

**Affiliations:** 1 Department of Experimental Immunology, Amsterdam, the Netherlands; 2 Renal Transplant Unit, Division of Internal Medicine, Academic Medical Center, Amsterdam, the Netherlands; 3 Sanquin Blood Supply Foundation and Landsteiner laboratory, Amsterdam, the Netherlands; 4 Department of Medical Microbiology, Leiden University Medical Center, Leiden, the Netherlands; University of Basel, SWITZERLAND

## Abstract

Polyomavirus BK (BKPyV) frequently reactivates in immunosuppressed renal transplant recipients (RTRs) and may lead to graft loss due to BKPyV-induced interstitial nephritis (BKVN). Little is known on the differentiation of CD8^+^ T cells targeting BKPyV in RTRs. Here we investigated whether BKPyV-specific CD8^+^ T cell differentiation differs in RTRs with varying degrees of BKPyV reactivation and/or BKVN.

Using combinatorial encoding with tetramers carrying BKPyV major capsid protein (VP1) and large T antigen protein (LTAG) epitopes, we investigated CD8^+^ T cell responses to BKPyV in longitudinally obtained PBMC samples from 46 HLA-A02-positive RTRs and 20 healthy adults. We were also able to isolate BKPyV-specific CD8^+^ T cells from five renal allografts, two of which were affected by BKVN.

Before transplantation, BKPyV-specific CD8^+^ T cells targeting VP1 and LTAG epitopes appeared predominantly as central-memory and CD27^+^/CD28^+^ effector-memory (T_EM_), and naïve-like PD-1-expressing cells, respectively. After viral reactivation, BKPyV-specific CD8^+^ T cells assumed CD28^−^ T_EM_ and T_EM_RA states in patients who were able to control BKPyV, whereas differentiation lagged behind in patients with severe viral reactivation or BKVN. Furthermore, VP1-specific CD69^+^/CD103^+^ tissue-resident memory (T_RM_) cells accumulated in BKVN-affected allografts but lacked signs of effector differentiation. In contrast, granzyme B-expressing effector cells were detected in allografts not affected by BKVN.

In conclusion, effector-memory differentiation of BKPyV-specific CD8^+^ T cells in patients with high viral load or BKVN is impaired. Further characterization of the specific mechanisms behind this altered cellular differentiation is necessary to develop therapies that can prevent the emergence of BKVN.

## Author Summary

In immunosuppressed renal transplant recipients (RTRs), BKPyV frequently reactivates from latency and may cause severe interstitial nephritis in the allograft (BKVN). Not only is there no effective treatment, it also not understood why BKVN arises in some RTRs but not in all. In the current study we investigated populations of CD8^+^ T cells targeting epitopes from structural and non-structural BKPyV proteins in RTRs over the course of transplantation. In contrast to RTRs who suffered from self-limiting reactivation of BKPyV, patients who developed severe viral reactivation and BKVN were found to have BKPyV-specific CD8^+^ T cells which did not, or less often differentiate into CD28^−^ effector-memory cells during viral reactivation. Moreover, virus-specific CD8^+^ T cell activation and differentiation was not only impaired in the circulation, but possibly also in BKVN-affected renal allografts. In contrast to the CD8^+^ T cells in kidneys from three patients who did not develop BKVN, T cells in two BKVN-affected kidneys did not display typical cytotoxic effector traits. These findings suggest that impaired BKPyV-specific CD8^+^ T cell maturation in response to viral reactivation, possibly owing to inter-individual differences in sensitivity to immunosuppressive medication or to certain viral quasispecies, underlies the emergence of severe viral reactivation and BKVN.

## Introduction

Polyomavirus BK (BKPyV) establishes a mode of latent infection in the vast majority of the general, immunocompetent population [1, 2]. However, in immunosuppressed renal transplant recipients (RTRs), BKPyV can escape the weakened immunological response leading to reactivation in up to 60% of the patients. In as much as 10% of these reactivations, the virus causes a severe interstitial nephritis (BKVN) in the allograft that is associated with graft loss [3, 4]. Until now, the only effective treatment option for BKPyV reactivation following renal transplantation involves tapering of the immunosuppressive drug therapy, allowing the patient’s immune system to recover and overcome the virus. However, this also increases the chance on allograft rejection [3, 4].

For these reasons, effective and more specific treatment strategies are urgently needed. It is here that modern immunotherapies, such as adoptive transfer of virus-specific T cells, come into view. Recently, it was shown that BKPyV reactivation occurs concomitantly to a loss of polyfunctional T cells specifically targeting BKPyV epitopes, emphasizing the importance of T cells for effective immunological control of this virus [5–7]. T cell populations specific for BKPyV can be expanded *in vitro* and may then theoretically be used to treat BKPyV reactivation [8]. However, because each human virus triggers the formation of a specialized subset of T cells, carrying a distinct armamentarium to combat the respective virus [9], it is essential to understand what type of T cells confers protection against BKPyV.

Previously, we used BKPyV virion protein 1 (VP1) peptide-loaded HLA A02-restricted tetramers to determine the phenotype and function of VP1-specific CD8^+^ T cells in the circulation of healthy individuals. We found that these cells largely exist in a central-memory (T_CM_) or early-differentiated state [10], a phenotype that was recently associated with stem cell-like properties [11]. However, in healthy individuals BKPyV-specific T cells may seldom encounter their cognate antigen [12], whereas in RTRs BKPyV frequently reactivates, thus exposing the host’s T cells to substantial amounts of antigen and inflammation. Because of their specific capacity to detect and control intracellular pathology, as caused by viruses, we here investigated the phenotypic and functional differentiation of BKPyV VP1- and large T antigen (LTAG)-specific CD8^+^ T cells in the circulation of RTRs suffering from various degrees of BKPyV reactivation over the course of transplantation. In addition, we characterized BKPyV-specific T cells obtained from the allograft of some patients. Using this approach we aimed to identify whether differences in clinical outcome of BKPyV-infection are associated with altered differentiation pathways and/or effector functions of CD8^+^ T cells targeting this virus.

Using combinatorial encoding with six different HLA A02-restricted tetramers we confirmed that VP1-specific cells before transplantation mainly exist in a central-memory (T_CM_) or early-differentiated effector-memory (T_EM_) state, whereas LTAG-specific CD8^+^ T cells unexpectedly exhibit a naïve-like phenotype with frequent expression of PD-1. After transplantation, both VP1 and LTAG-specific cells showed CD28^−^ T_EM_ differentiation, sometimes with CD45RA re-expression (T_EM_RA). This mainly occurred in RTRs with low or undetectable viral load but not in patients with high viral load and/or BKVN. Within the renal allograft of two BKVN patients, we detected a high frequency of CD69/CD103-expressing tissue-resident BKPyV VP1-specific memory cells that, in contrast to the CD69/CD103-negative recirculating BKPyV-specific cells in kidneys from non-BKVN-affected patients, did not express granzyme B.

## Results

### Patients and virology

We included longitudinally obtained samples from 46 HLA-A02-positive RTRs: 21 in whom BKPyV replication had not been observed in the first year after transplantation (not-reactivating or NR patients), 11 RTRs in whom BKPyV had reactivated with a peak viral load below 1*10^4^ copies/ml (R^low^ patients), 6 RTRs showing BKPyV reactivation with a peak viral load higher than 10^4^ copies/ml (R^high^ patients), and 8 RTRs with peak viral load higher than 10^4^ copies/ml and biopsy-proven BKVN (BKVN patients). Samples from 20 HLA A02-positive healthy individuals served as a control. When comparing all study groups containing RTRs, there was a statistically significant difference in overall HLA mismatches that derived from a high total number of mismatches in the NR patients. Also, donor age was greater in the R^low^ patients when compared to BKVN patients. Finally, estimated glomerular filtration rates were significantly lower in R^high^ patients when compared to NR patients (Table 1).

**Table 1:** Patient characteristics.

	Low peak viral load [VL<10e4 c/ml]	High peak viral load [VL>10e4 c/ml]	BKVN	No BKPyV reactivation [NR]	Healthy individuals	P-value
**Recipient**						
Number	11	6	8	21	20	
Age (median (yr), quartiles)	63 (53–66)	62 (54–65)	58 (52–62)	56 (47–65)	-	0.79
Gender (% male)	45.5%	66.7%	50%	61.9%	-	
Pre-transplant CMV status (% positive)	45.5%	50%	87.5%	85.7%	-	
Pre-transplant EBV status (% positive)	90.9%	100%	100%	100%	-	
**Donor**						
Age (median (yr), quartiles)	65 (63–70)°	55 (46–61)	47 (39–58)°	53 (46–61)	-	0.10
Gender (% male)	72.7%	50%	75%	52.4%	-	
Deceased donor (%)	45.5%	50%	75%	71.4%	-	
**HLA mismatches** (median, quartiles)						
HLA A	1 (1–1)	1 (0.3–1)	1 (0–1)	1 (0–1)	-	0.28
HLA B	1 (1–2)	1 (1–1.8)	1 (1–1.3)	1 (1–2)	-	0.89
HLA DR	1 (0.5–1.5)	0.5 (0–1)	1 (0–1)	1 (0–2)	-	0.57
HLA A/B/DR	4 (2.5–4.0)	2.5 (2–3.8)#	2 (1.8–3)	3 (2–5)#	-	0.001
**BKV infection**						
Time point of reactivation (weeks post Tx) median [IQR]	28 (27–33)* °	20 (15–22)*	20 (13–25)°	-	-	0.004
Duration to peak viral load (weeks post Tx) median [IQR]	28 (28–43)	26 (22–26)	38 (29–49)	-	-	0.117
BKV DNA load at peak (median (*10e3 copies/ml, blood), quartiles)	2 (0.5–2.9)* °	52 (16–206)* ^▫^	422 (144–985)° ^▫^	-	-	<0.001
**Graft outcome**						
Delayed Graft Function (% present)^2^	27.3%	50%	50%	47.6%	-	
Cold ischemia (median (h), quartiles)	3 (2–14)	7 (2–14)	12 (5–18)	13 (3–18)	-	0.26
Rejection episode < 1yr (% present)^1^	9.1%	16.7%	12.5%	4.8%	-	
Rejection episode > 1yr (% present)^1^	10%	0%	12.5%	10%	-	
**eGFR** ^3^ (median, (mL/min per 1.73 m^2^), quartiles)^3^					-	
1 year post TX	35.0 (29.0–43.5)	44.0 (36.8–50.0)	31.0 (29.0–43.0)	45.0 (38.5–46.0)	-	0.60
2 years post TX	35.0 (32.0–39.0)	52.0 (40.0–52.0)	34.0 (25.5–38.8)^φ^	45.5 (41.5–50.0)^φ^	-	0.052

^1^ T-cell-mediated rejection and/ or Antibody-mediated rejection

^2^ Delayed graft function was defined as the need for dialysis in the first week following transplantation

^3^ Estimated GFR (eGFR) was calculated using the abbreviated MDRD formula published by Levey et al. [14]: eGFR = 175 x (P_cr_ ÷ 88.4)^− 1.154^ x age^− 0.203^ x 0.742 [if female] x 1.210 [if black]

(*) R^low^ vs R^high^;

(°) R^low^ vs BKVN;

(^▫^) R^high^ vs BKVN;

(^#^) R^high^ vs NR;

(^φ^) BKVN vs NR.

From five other patients who underwent a graft biopsy because of deterioration in renal allograft function during active BKPyV-infection, we obtained graft-eluted cells. Histological examination revealed BKVN in 2 of them, and no BKPyV infection in the other three patients. All grafts contained various degrees of interstitial fibrosis, tubular atrophy and cellular infiltrates.

**Fig 1:**
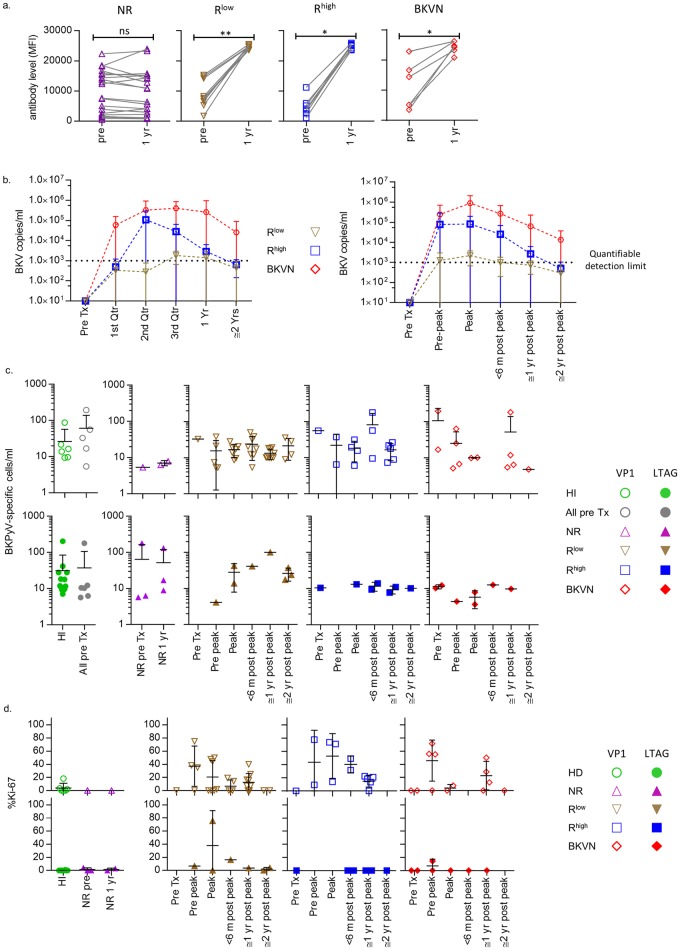
(**A**) anti-VP1 antibody levels in NR, R^low^, R^high^ and BKVN patients shortly before transplantation and one year after transplantation. (**B**) Viral load during follow-up of R^low^, R^high^ and BKVN patients (left panel) and viral load plotted against the peak viral load (right panel). (**C**) From left to right: Population sizes of VP1- (open symbols) and LTAG-specific (closed symbols) CD8^+^ T cells detected in healthy individuals, in all RTRs before transplantation, in NR patients before—and one year after transplantation and in the R^low^, R^high^ and BKVN during follow-up. (**D**) Expression frequency of Ki-67 by VP1- (open symbols) and LTAG-specific (closed symbols) CD8^+^ T cells in healthy individuals, in NR patients before—and one year after transplantation and in the R^low^, R^high^ and BKVN RTRs during follow-up.

Serological assessment showed the presence of anti-BKPyV antibodies in all patients before transplantation. Antibody titres increased significantly in the first year after transplantation in all RTRs in whom BK viremia was detected, but not in the NR patients (Fig 1A). Therefore, the rise in antibody titres is a reflection of viral reactivation as measured in the circulation but does not necessarily seems to prevent the reactivation as was shown previously [5, 13]. Peak viral load in RTRs were detected most often in the second and third quarter of the first year post transplantation (Fig 1B). The viral load in the R^low^ patients had dropped close to the quantifiable detection threshold of 1000 copies/ml already at the ≤6 months post peak viral load points. In the R^high^ patients, this did not occur until somewhere in between the ≤1 year and ≤ 2 year post peak viral load time points. BKVN patients did not drop below this threshold during follow-up (Fig 1B). In response to detection of BKPyV viremia, the dosage of immunosuppressive drugs was carefully diminished, aimed at decreasing the BKPyV-load and preserving renal allograft function. First, the dose of mycophenolate mofetil was tapered in steps of 250 to 500 mg per 2 weeks, followed by decreasing the dose of tacrolimus by 0.5 to 2 mg per 2 weeks.

### Detection of BKPyV-specific CD8^+^ T cells in healthy individuals and in RTRs before and following transplantation

Previously, BKPyV-specific CD8^+^ T cells were shown to be present in the circulation of both healthy individuals and RTRs at extremely low frequencies [10, 15–18]. To enhance the sensitivity and specificity of detection of BKPyV-specific CD8^+^ T cells, we here used combinatorial encoding of HLA-A02 tetramers loaded with two different immunodominant BKPyV VP1 peptides and one immunodominant LTAG peptide (S1A Fig). Using this technique, and staining a large number of PBMCs (up to 12*10^6^ PBMCs per sample), we detected BKPyV VP1-specific CD8^+^ T cells in 6 out of 20 healthy individuals, and in 2 of 21 NR patients; 8 of 11 R^low^ patients; 6 of 6 R^high^ patients; and in 5 of 8 BKVN patients at some time point(s) during follow-up. We detected LTAG-specific cells in 12 of 20 healthy individuals, and in 4 of 21 NR patients; in 2 of 11 R^low^ patients, in 2 of 6 R^high^ patients and in 4 of 8 BKVN patients during follow-up (S2 Fig). In RTRs, both VP1 and LTAG-specific cells were detected more frequently during viremia. Expansion of BKPyV-specific CD8^+^ T cell populations occurred in some individuals after transplantation, but not in all patients (Fig 1C). This was corroborated by a rise in Ki-67 expression after transplantation, particularly by the VP1-specific cells, indicating active cell proliferation. Ki-67^+^ expressing cells were detected neither in the samples from the NR patients, nor in those from the healthy individuals (Fig 1D).

### The differentiation status of BKPyV-specific CD8^+^ T cells in RTRs before transplantation is similar to that in healthy individuals

**Fig 2:**
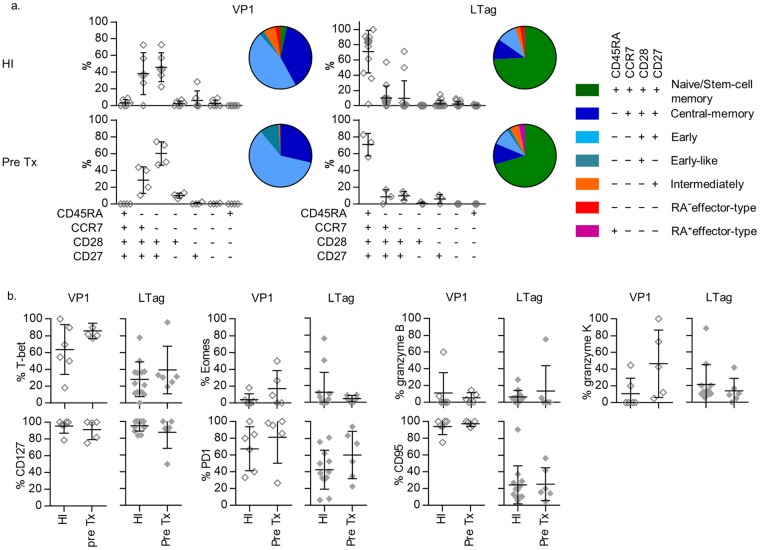
(**A**) Scatter plots and pie charts showing the distribution of the seven largest CD45RA/CCR7/CD28/CD27-defined human CD8^+^ T cell populations, as described previously [21], amongst VP1- (first column) and LTAG-specific (second column) CD8^+^ T cell populations detected in healthy individuals (first row) and all RTRs (second row) before transplantation. (**B**) from left to right the expression of T-bet, Eomes, granzyme B, granzyme K (first row) and IL-7Rα (CD127), PD-1 and CD95 (second row) by VP1- (open symbols) and LTAG-specific (closed symbols) CD8^+^ T cells detected in healthy individuals and in all RTRs before transplantation.

Using multichannel flowcytometry, we determined the expression of various molecules characteristic for T cell differentiation and function (S1B Fig). Previously, we found that circulating BKPyV VP1-specific CD8^+^ T cells in healthy individuals were predominantly T_CM_ cells (CD45RA^−^CCR7^+^CD27^+^) or early-differentiated T_EM_ (CD45RA^−^CCR7^−^CD27^+^) cells [10]. In the current study, adding the expression of CD28 to the classification, we confirmed these findings (Fig 2A).

Both LTAG and VP1-specific CD8^+^ T cells circulating in RTRs before transplantation showed similar phenotypes as in healthy individuals (Fig 2A). Comparison of LTAG and VP1-specific CD8^+^ T cells, however, revealed substantial differences in both healthy individuals and RTRs, with the LTAG-specific CD8^+^ T cells displaying a predominant CD45RA^+^CCR7^+^CD28^+^CD27^+^ surface phenotype (Fig 2A). This phenotype may define antigen-inexperienced T cells, but also a subset of very early differentiated antigen-experienced CD8^+^ T cells with stem-cell-like traits, that, amongst others, is defined by expression of the tumour necrosis factor receptor family member CD95 (FAS receptor) [19, 20]. However, only about 16% of LTAG-specific CD8^+^ T cells with a “naïve” CD45RA^+^CCR7^+^CD28^+^CD27^+^ phenotype expressed CD95, which equalled the CD95 expression on the total population of CD45RA^+^CCR7^+^CD28^+^CD27^+^ CD8^+^ T cells (S3 Fig). Thus, based on this surface marker, only a fraction of LTAG-specific cells could be assigned as typical stem-cell memory cells. Importantly, the LTAG-specific cells were significantly enriched for the expression of PD-1 when compared to the total naïve CD8^+^ T cell pool (S3 Fig) suggesting that they have indeed been stimulated by antigen.

In addition, no major differences were found between the BKPyV-specific CD8^+^ T cells of patients just before renal transplantation and healthy control individuals regarding other immunological characteristics of BKPyV-specific cells like their T-bet- or Eomes expression; expression of granzyme B or granzyme K, and IL-7Rα (CD127), PD1, or CD95 (Fig 2).

### BKPyV-specific CD8^+^ T cell effector-memory differentiation is impaired in renal transplant recipients with high viral load and BKVN

**Fig 3:**
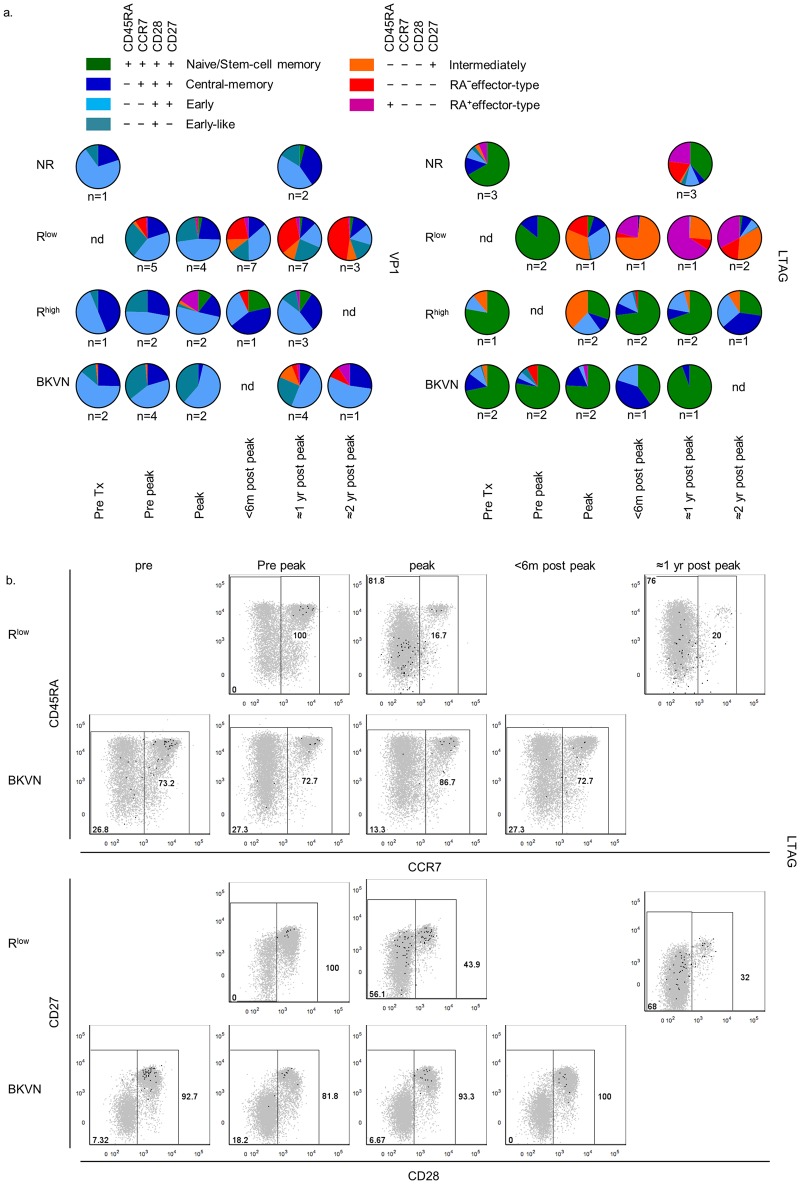
(**A**) Pie charts depicting the distribution of the seven largest CD45RA/CCR7/CD28/CD27-defined human CD8^+^ T cell populations, as described previously [21], amongst VP1- (left panel) and LTAG-specific (right panel) CD8^+^ T cell populations detected in NR, R^low^, R^high^ and BKVN RTRs during follow-up. (**B**) Representative dot plot overlays showing the fluorescence intensities of CD45RA, CCR7, CD28 and CD27 with the total CD8^+^ T cell events shown in grey and LTAG-specific events in black from one R^low^ patient (upper row) and one BKVN patient (lower row) during follow-up.

During BKPyV reactivation, the composition of both VP1- and LTAG-specific CD8^+^ T cell populations changed, as shown in Fig 3A and S4 Fig. The most profound changes were noted in the R^low^ patients, in whom substantial proportions of normally cytotoxic intermediately-differentiated (CD45RA^−^CCR7^−^CD28^−^CD27^+^), CD45RA^−^ effector-type (CD45RA^−^CCR7^−^CD28^−^CD27^−^) T_EM_ and T_EM_RA (CD45RA^+^CCR7^−^CD28^−^CD27) CD8^+^ T cell subsets specific for either VP1 or LTAG became detectable during and after the time point of peak viral load. In the R^high^ and BKVN group, these subsets were also formed amongst the VP1-specific CD8^+^ T cells but later in time and in smaller proportions. CD28^−^ T_EM_ subsets also emerged amongst LTAG-specific populations, but primarily at the moment of peak viral load in the R^high^, after which their sizes diminished during the later time points. CD28^−^ T_EM_ differentiation was seldom observed in the BKVN patients. Differentiation had also occurred within the LTAG-specific cell-populations from NR patients at one year post-transplantation (Fig 3 and S4 Fig).

### The frequency of T-bet and Eomes-expressing LTAG-specific CD8^+^ T cells is highest in patients with low BK viral load

Recently, we found that the expression levels of T-bet and Eomes, master transcriptional regulators of type 1 (cytotoxic) T cell differentiation, are strong indicators of the degree of CD8^+^ T cell differentiation [21]. We also showed that BKPyV VP1-specific CD8^+^ T cells circulating in healthy individuals mostly express low or intermediate levels of T-bet, whereas they lack expression of Eomes [10]. Here, we studied whether the expression of T-bet and Eomes was influenced by the BK viremia occurring in RTRs.

**Fig 4:**
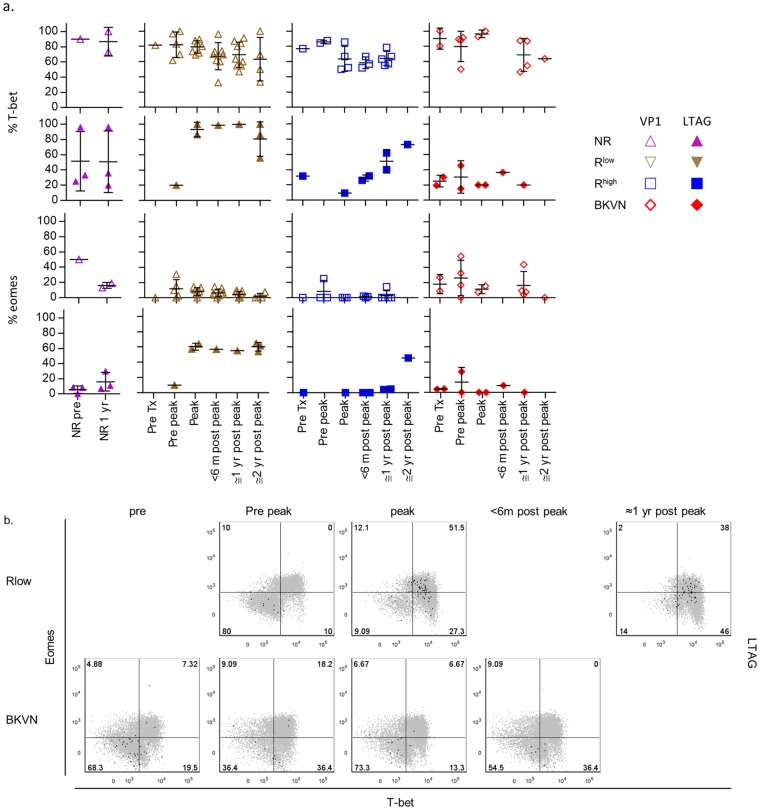
(**A**) Scatter plots showing the expression frequencies of T-bet (upper panel) and Eomes (lower panel) by VP1- (open symbols) and LTAG-specific (closed symbols) CD8^+^ T cell populations detected in NR patients before—and one year after transplantation, and in the R^low^, R^high^ and BKVN RTRs during follow-up. (**B**) Representative dot plot overlays showing the fluorescence intensities of T-bet and Eomes with the total CD8^+^ T cell events shown in grey and LTAG-specific events in black from one R^low^ patient (upper row) and one BKVN patient (lower row) during follow-up.

Fig 4A shows that at all time points and in each patient group, VP1- and LTAG-specific cells expressed significantly more T-bet than Eomes. The frequency of T-bet- and Eomes-expressing VP1-specific cells was comparable between the different study groups. Although referring to data from only six patients, the frequency of both T-bet and Eomes-expressing LTAG-specific CD8^+^ T cells appeared to be higher in the R^low^ patients than in the other study groups. This is also illustrated by Fig 4B, which shows two representative patients from the R^low^-, respectively BKVN group. Remarkably, despite the clear CD28^−^ T_EM_ differentiation detected in the LTAG-specific CD8^+^ T cells from NR patients around the first year after transplantation (Fig 3A), these populations did not contain increased frequencies of T-bet- and Eomes- expression at that time point (Fig 4A).

### During BKPyV-replication, IL-7Rα expression on LTAG-specific CD8^+^ T cells in patients with low BK viral load is down regulated

**Fig 5:**
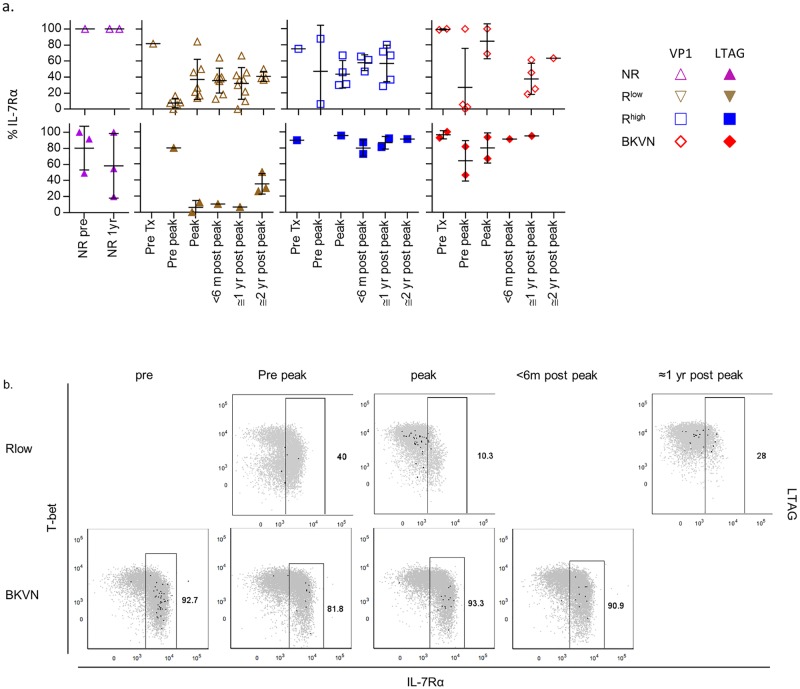
(**A**) Scatter plots showing the expression frequencies of IL-7Rα by VP1- (open symbols) and LTAG-specific (closed symbols) CD8^+^ T cell populations detected in NR patients before—and one year after transplantation, and in the R^low^, R^high^ and BKVN RTRs during follow-up. (**B**) Representative dot plot overlays showing the fluorescence intensities of T-bet and IL-7Rα with the total CD8^+^ T cell events shown in grey and LTAG-specific events in black from one R^low^ patient (upper row) and one BKVN patient (lower row) during follow-up.

The cytokine IL-7 is important for T cell homeostasis in the absence of antigen and inflammation and IL-7Rα expression is rapidly lost following T cell receptor-dependent activation [22]. As described previously, nearly all VP1-specific cells in healthy individuals expressed IL-7Rα, further suggesting that these cells infrequently encounter their antigen (Fig 2) [10]. As shown above, we found similar data for the LTAG-specific cells in healthy individuals and in patients just before renal transplantation (Fig 2B). IL-7Rα was also expressed on the majority of BKPyV-specific CD8^+^ T cells in NR-patients, R^high^ and BKVN patients (Fig 5A). In sharp contrast, IL-7Rα expression in the R^low^ patients was clearly downregulated during BKPyV-reactivation, especially on the LTAG-specific cell populations, as is also illustrated by two representative patients from the R^low^—, respectively the BKVN group (Fig 5B).

### Functional characteristics of BKPyV-specific CD8^+^ T cells

Next, we studied functional properties of BKPyV-specific CD8^+^ T cells, viz. their cytotoxic capacity as judged by both the presence of the serine proteases granzyme K and granzyme B, expression of the degranulation marker CD107a and their cytokine-producing capacity.

**Fig 6:**
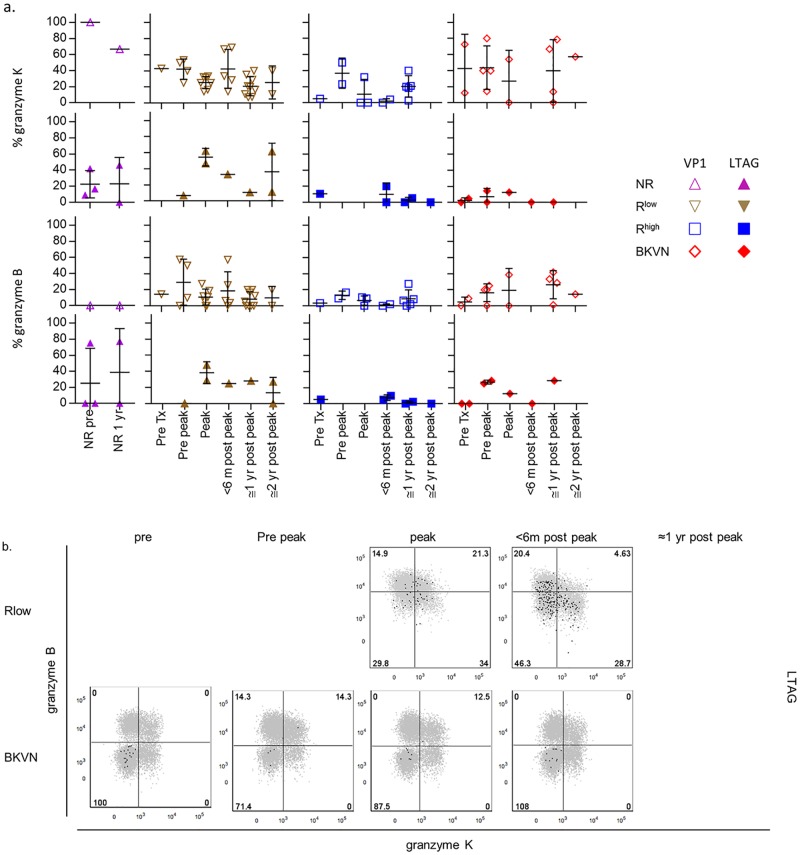
(**A**) Scatter plots showing the expression frequencies of granzyme K (upper panel) and granzyme B (lower panel) by VP1- (open symbols) and LTAG-specific (closed symbols) CD8^+^ T cell populations detected in NR patients before—and one year after transplantation, and in the R^low^, R^high^ and BKVN RTRs during follow-up. (**B**) Representative dot plot overlays showing the fluorescence intensities of granzyme K and granzyme B with the total CD8^+^ T cell events shown in grey and LTAG-specific events in black from one R^low^ patient (upper row) and one BKVN patient (lower row) during follow-up.

**Fig 7:**
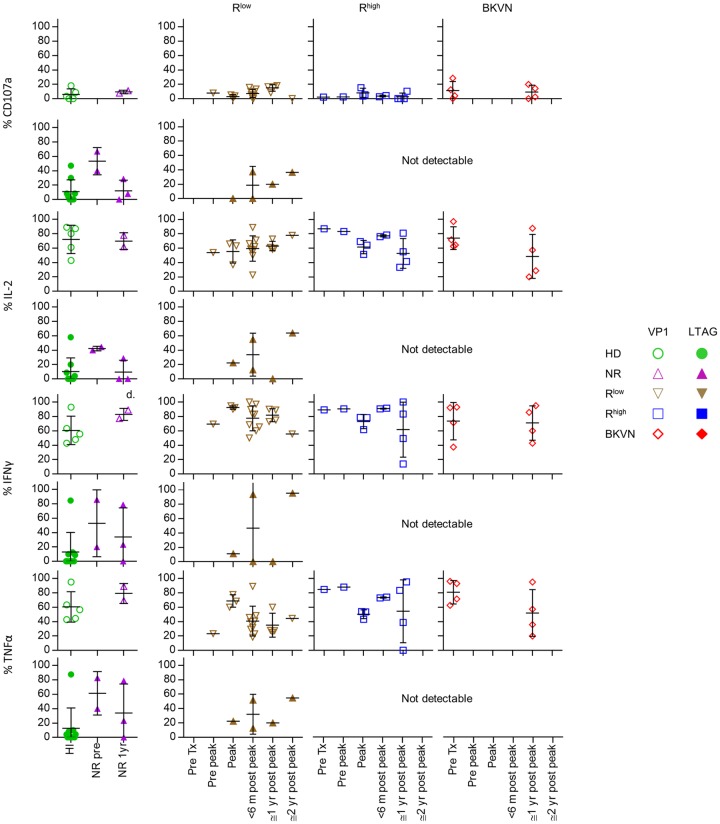
Scatter plots showing the production of CD107a (first row), IL-2 (second row), IFNγ (third row) and TNFα (last row) by VP1- (open symbols) and LTAG-specific (closed symbols) CD8^+^ T cell populations detected after stimulation in vitro in healthy individuals, NR patients before—and one year after transplantation, and in the R^low^, R^high^ and BKVN RTRs during follow-up.

Previously, we found that a small number of BKPyV VP1-specific CD8^+^ T cells in healthy individuals expressed granzyme K and/or B, which we confirmed in the present study (Fig 2) [10]. Despite the CD28^−^ T_EM_ differentiation occurring after BKPyV reactivation, particularly in the R^low^ group, no clear differences in granzyme expression were observed at any time-point between these and other patients (Fig 6). As a marker for degranulation, we studied the surface expression of CD107a on BKPyV-specific CD8^+^ T cells after stimulation in vitro. Fig 7 shows in all groups at all time points a rather low frequency of CD107a^+^ cells, suggesting minimal degranulation of these cells, at least in the peripheral circulation.

The cytokine production capacity of the different BKPyV-specific CD8^+^ T cell populations was tested by stimulating PBMC with PMA/ionomycin, followed by visualization of the BKPyV-specific CD8^+^ T cells using combinatorial encoding with tetramers. This approach is hindered by downregulation of the T cell receptor upon T cell activation. For unknown reasons, this particularly affected the LTAG-specific cells in the R^high^ and BKVN patients. As such, we were unable to detect sufficient LTAG-specific cells in these patient groups for analysis. In the R^low^ group, where LTAG-specific cells were still detectable after stimulation, we observed that a modest proportion produced IL-2, TNFα and INFγ (Fig 7 and S5 Fig).

Previously, we found that the majority of VP1-specific CD8^+^ T cells in healthy individuals produced combinations of three cytokines, most commonly IL-2, INFγ and TNFα [10]. This was confirmed in the present study, and was also observed in patients before renal transplantation and thereafter, irrespective of detectable BKPyV reactivation (Fig 7). No major differences in cytokine production capacity of VP1-specific cells were observed during follow-up.

### Tissue-resident memory CD8^+^ T cells directed against the BKPyV VP1-epitope accumulate in the renal allograft from patients with BKVN

**Fig 8:**
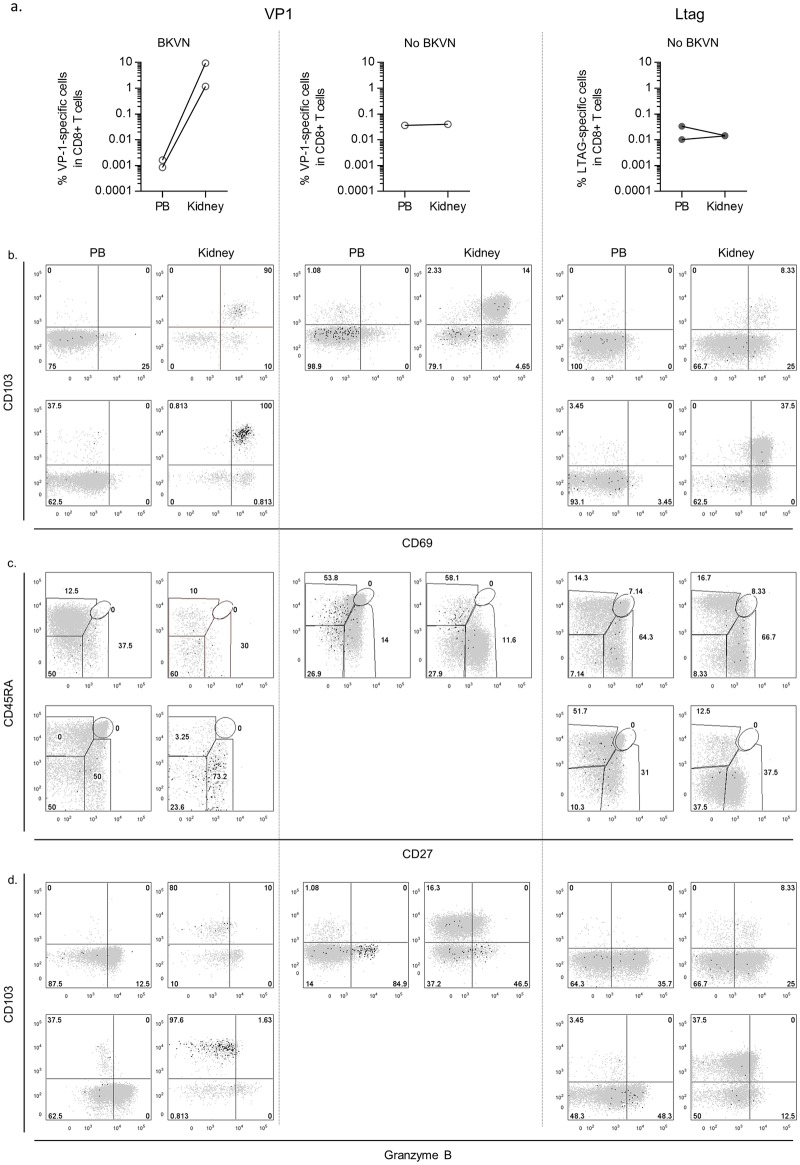
(**A**) Line graphs showing the paired percentages of BKPyV VP1- and LTAG-specific CD8^+^ T cells amongst the total CD8^+^ T cell pool in the peripheral blood (PB) and in the kidney for 2 BKVN patients (first column) and three other RTRs (middle and right columns) (**B**) Dot plot overlays showing the fluorescence intensities of CD013 and CD69, and of (**C**) CD45RA and CD27, and (**D**) CD103 and granzyme B in the PB and in the kidney.

Because BKPyV nephropathy is the final consequence of uncontrolled BKPyV-replication in the kidney allograft, we studied the presence of BKPyV-specific CD8^+^ T cells within the graft of two patients and compared them to their peripheral blood counterparts. As a control, we studied graft-eluted cells from three RTRs without BKVN. In the two BKVN grafts, we detected only VP1-specific CD8^+^ T cells, whereas in the three non-BKVN-affected grafts we detected one VP1- and two LTAG-specific populations. We found that in the BKVN grafts the VP1-specific T cells were about 10e5 times enriched when compared to the peripheral blood compartment. In contrast, the frequencies of the one VP1-specific population and two LTAG-specific populations that we detected in the non-BKVN allografts, were similar to those in the paired peripheral blood samples (Fig 8A **)**.

Tissue-resident memory T-cells (T_RM_) are characterized by expression of CD69 and CD103 [23, 24], both molecules ensuring that T_RM_ populations are retained in the respective tissue and that they do not re-enter the circulation [25]. In the two patients with BKVN, most of the graft-eluted VP1-specific CD8^+^ T cells expressed both CD69 and CD103, designating them as T_RM_ cells (Fig 8B). In contrast, in patients without BKVN, a minority of the BKPyV-specific CD8 T cells stained double-positive for these markers. The graft-eluted VP1-specific CD8^+^ T cells from the two BKVN patients were comparable to those in peripheral blood, showing a CD45RA^−^CD27^+/−^ T_EM_ phenotype (Fig 8C). Both VP1- and LTAG-specific graft-eluted cells from patients without BKVN were also quite similar to their PB counterparts but showed a more advanced differentiation state, bearing a CD27^−^ T_EM_ or T_EM_RA phenotype. The accumulated VP1-specific CD8^+^ T cells in the two BKVN-affected kidneys contained very few granzyme B positive cells. In contrast, a considerable proportion of the BKPyV-specific CD8^+^ T cells in the non-BKVN-kidneys expressed this serine protease, although the percentage was lower than in the PB compartment (Fig 8D).

## Discussion

Here, we document that in renal transplant patients with high viral load and/or BKVN, the effector-memory differentiation of circulatory BKPyV VP1- and LTAG-specific CD8^+^ T cells is distinct from that in patients with low viral load. VP1-specific CD8^+^ T cells collected before transplantation started off with a T_CM_ or early-differentiated T_EM_ phenotype, whereas the LTAG-specific cells curiously primarily displayed a naïve-like phenotype. Nevertheless, following transplantation and viral reactivation in the R^low^ patients, both VP1- and LTAG-specific populations differentiated into CD28^−^ T_EM_ cells, with LTAG-specific cells even acquiring the T_EM_RA state. In the R^high^ and BKVN patients, VP1- and LTAG-specific CD8^+^ T cells instead generally persisted in their T_CM_ and CD28^+^CD27^+^ T_EM_ differentiation state.

In line with this, the frequency of circulating T-bet and Eomes-expressing LTAG-specific cells was highest in patients with low viral replication. Furthermore, the BKPyV-specific CD8^+^ T cells in R^low^ patients downregulated their expression of IL-7Rα, emphasizing the activation of these cells. Despite these dissimilarities in differentiation patterns, the BKPyV-specific cells in the distinct patient groups expressed similar but low levels of granzyme K and B. Also, we did not find any difference between the groups in cytokine production by the BKPyV-specific CD8^+^ T cells, which were polyfunctional as we showed before. Because we found no differences in properties of BKPyV specific CD8^+^ T cells between healthy individuals and patients shortly before transplantation, possible effects exerted by the uremic state or by any drug medication at present or in the past seem not to be involved.

When compared to human cytomegalovirus (hCMV) or Epstein-Barr virus (EBV)-specific CD8^+^ T cells, the frequencies of BKPyV-specific cells in the circulation are very low, making them difficult to detect [10, 15–18, 26]. Schachtner et al. used in vitro stimulation with overlapping BKPyV peptide pools in an Interferon-γ Elispot assay, and showed that the overall BKPyV-specific CD4^+^ and CD8^+^ T cell response was significantly delayed in patients who developed BKVN [5]. The same group recently demonstrated that this delay concerns mainly the T cell response targeting LTAG epitopes [6], which is in line with the data presented here. Schaenman et al. recently also reported impaired BKPyV-specific CD8^+^ and CD4^+^ T cell responses in patients with severe reactivation of BKPyV. However, in contrast to the findings presented in the current manuscript, this group detected a particularly frequent expression of CD107a by BKPyV-specific CD8^+^ T cells [7]. We think that this results from much longer T cell stimulation *in vitro*, which was applied to assay T cell cytokine/CD107a expression capacity. We would like to emphasize that in vitro stimulation with peptide and co-stimulation for several hours will significantly alter the phenotype of T cells. For example CCR7, IL-7Rα and CD62L expression rapidly disappears from the cell surface after stimulation [22, 27, 28]. Also, in vitro T cell activation results in the induction of T-bet and Eomes expression, which directly induce expression of molecules like granzyme B, interferon-γ, CD122 and IL-15Rα [29–36]. This does not occur when using tetramers to isolate T cells if done in the proper conditions, viz. brief period of staining in the absence of co-stimulation, at a low temperature. The low expression frequency of CD107a, as detected in our current study, is in line with the low expression frequency of granzyme B by the BKPyV-specific CD8^+^ T cells, since both molecules are located in the same cytotoxic granules [37]. However, whereas tetramers are well fit to determine the phenotype and functional properties of antigen-specific T cells directly ex-vivo, they cannot visualize the total number of virus-specific T cells active against a given antigen, as can be done with overlapping peptide pool stimulation assays, owing to the epitope restrictions of the tetramers.

The naïve-like LTAG cells detected prior to transplantation expressed PD-1 significantly more often when compared to the total CD45RA/CCR7/CD28/CD27 CD8^+^ T cell population. Apart from being a marker of functional exhaustion, PD-1 is also recruited into the immunological synapse upon T cell activation [38, 39]. Therefore, this naïve-like state may represent a subset of antigen-experienced T cells in a very early differentiation state, close to the CD95-expressing naïve-like population of stem-cell memory cells that was described recently [19, 20].

In view of the low percentage of granzyme-expressing cells, it may therefore well be that the normal immunological control of BKPyV by CD8^+^ T cells is not exerted by granzyme K or B. For example, human CMV-specific CD8^+^ T cells highly express granzyme B and T-bet. Instead, CD8^+^ T cells targeting EBV epitopes, primarily express granzyme K and Eomes, suggesting that each virus is controlled by a distinct type of CD8^+^ T cell equipped with a specific armamentarium [21, 40–44]. Therefore, CD8^+^ T cells may also have adopted a distinct strategy to control BKPyV, especially considering the long relationship between man and this virus [45]. Given the polyfunctionality with regard to cytokine production, BKPyV-specific CD8^+^ T cells may rely much more on production of typical cytokines to control BKPyV proliferation than on exerting cytotoxicity against infected cells.

It is important to mention that we only investigated the immunodominant HLA-A02-restricted T cell response in this study. Whilst this was done because this is the most abundant HLA class I molecule expressed by the general Western population, immunodominant BKPyV T cell responses indeed also occur via other HLA class I molecules as shown recently by Cioni et al. [46]. Furthermore, different viral proteins can trigger different types of T cells, amongst which possibly cells with immunomodulatory function.

One should also consider that the mechanism by which viral control is executed, may not be reflected by T cells located in the peripheral blood compartment. Indeed, the epicentre of BKPyV infection and inflammation is located within the renal allograft and not in the circulation. In the two patients with BKVN, from whom we obtained graft-eluted cells, the frequency of VP1-specific CD8^+^ T cells in the graft was indeed much higher than in their paired peripheral blood samples, suggesting sequestration of virus specific cells within the allograft. The majority of these graft-eluted cells consisted of CD69/CD103 double-positive T_RM_ cells [23]. Surprisingly, also here only very few of these cells expressed granzyme B. Considering the immunopathology in BKVN grafts, as evidenced by histological damage and deteriorated graft function, this large T_RM_ population appeared not capable to control the viral infection. In contrast, in patients without BKVN, only few BKPyV specific T_RM_ cells were detected in the graft, that apparently possibly contributed to local control of the virus.

Although more granzyme B-expressing cells were present than in the BKVN patients, they were mainly CD103-negative and their frequency was still lower than in the peripheral blood compartment. Probably, the intragraft BKPyV-specific CD8^+^ T-cells with the CD103-negative effector phenotype, are recirculating cells. In fact, in paired peripheral blood samples, similar phenotypes were found. Whether the T_RM_ cells originate from in situ differentiation of these recirculating effector cells, or vice versa, is unknown. Neither do we understand why so few effector cells were detected in the BKVN-allografts, and why the large population of T_RM_ cells in the BKVN-allografts failed to contain the infection. This situation is reminiscent of so-called tumour-infiltrating lymphocytes (TILs), which are in general dysfunctional [47]. By analogy with that, we suppose to name these cells as Virus-specific Tissue-Infiltrating Lymphocytes (V-TILs). Given the small sample size in the current study, further research into (BKPyV-specific) kidney-resident T cell memory populations is required.

Specific reasons for the impaired effector-memory differentiation of circulating BKPyV-specific CD8^+^ T cells in the patients with high viral loads / BKVN require further research. One possibility is that differentiation did occur, but was not measurable in the peripheral blood compartment due to retention of these cells in the tissue. Considering truly impaired differentiation, this may be the consequence of defective CD4^+^ helper cell function, insufficient costimulation, individual differences in susceptibility to immunosuppressive medication, or differences in the virulence of various BKPyV sub- or quasispecies. More knowledge on these possibilities, also on BKPyV-specific CD4^+^ T cell differentiation in these patients, is needed to better understand the disease process in order to develop effective BKPyV-directed immunotherapy in the future.

In conclusion, our findings show an impaired effector-memory differentiation program of BKPyV-specific CD8^+^ T cells in patients with severe BKPyV reactivation and/or BKVN. This offers an explanation for the pathogenesis of this clinical entity in RTRs, as well as a rationale for the potential effectiveness of immunotherapies to treat BKPyV reactivation in the future.

## Materials and Methods

### Subjects and study groups

From the cohort of renal transplant recipients (RTRs) who were transplanted at the Academic Medical Center (AMC, Amsterdam, The Netherlands) between 2008 and 2013, we selected 25 HLA-A02-positive patients, who experienced a reactivation of BKPyV-infection as demonstrated by a positive DNA real-time quantitative PCR (qPCR) in plasma within the first two years after a first transplantation. We included only HLA-A02-positive individuals in this study because this is the most ubiquitously expressed HLA subclass (~50%) by the Western population. BKPyV DNA was quantified before and at regular intervals of 3 months after transplantation, and more frequently when qPCR had become positive, or earlier when BKPyV reactivation was clinically suspected. Peripheral blood samples were collected at the same time points; mononuclear cells (PBMC) and sera or plasma samples were frozen.

Time points chosen for analyses comprise: pre-transplantation (pre Tx); the period prior to detection of the peak viral load (pre-peak); the moment of peak viral load; the period of the first 6 months after detection of the peak viral load (≤6 months post peak); the period from month 6 to month 12 after detection of the peak viral load (≤ 1 year post peak); and the period between the first year and the second year after detection of the peak viral load (≤2 years post peak). Data points of individual patients shown and analysed were the ones collected closest to t = 6 months post peak, t = 12 months post peak and t = 24 months post peak. The pre-peak time point was defined as the number of months from transplantation to peak viral load divided by two. For obvious reasons, these restrictions did not apply to the pre-transplantation samples and the peak viral load samples as these concerned single sampling moments. Each time frame holds no more than one data point from an individual patient. All other data points collected and measured during follow-up were excluded from the analyses and the graphs shown in this manuscript.

Immunosuppressive treatment included induction with CD25mAb (Basiliximab), and maintenance therapy, consisting of corticosteroids 10 mg/day orally, mycophenolate mofetil 2 gram/day and tacrolimus aimed at serum trough levels of 6–10 ng/ml. Exclusion criteria comprised previous transplantation, PRA > 5%, inadequate viral load monitoring frequency, inadequate sampling frequency and/or treatment with immunosuppressive medication other than the agents described above. From the same cohort of RTRs, 21 HLA-A02-positive patients were included in whom no BKPyV reactivation occurred. These patients were treated, monitored and sampled according to the same protocol. In addition, we isolated mononuclear cells from renal allograft tissue and paired peripheral blood of 5 RTRs. Two patients who underwent a graft biopsy because of deterioration in renal allograft function during active BKPyV-infection were diagnosed to have BKVN based on histological analysis and a positive SV40 staining. BKPyV was not actively replicating in the three other RTRs and histological signs of BKPyV infection were lacking. All grafts contained various degrees of interstitial fibrosis, tubular atrophy and cellular infiltrates. As a control, we also included PBMC isolated from 20 HLA-A02-positive buffy coats from healthy blood donors ranging between 18 and 64 years of age (Sanquin, Blood Supply, Amsterdam, the Netherlands, Table 1). For these latter subjects we could not obtain serum samples. We chose a viral load of 10e4 copies/ml as cut-off value between R^low^ and R^high^ patients, because it was previously proposed as a critical threshold for developing BKVN [48]. However, as opposed to the BKVN patients, we were unable to detect BKVN in the R^high^ patients by immunohistochemistry of their allograft biopsies.

### Ethics statement

The study was approved by the Medical Ethical Committee of the AMC, and written-informed consent was obtained from all patients in accordance with the Declaration of Helsinki.

### Isolation of mononuclear cells from peripheral blood and renal allograft tissue

PBMC were obtained using standard density gradient centrifugation and subsequently cryopreserved until the day of analysis [49]. Samples of human renal cortex were obtained from transplantectomies and renal allograft biopsies. Kidney mononuclear cells were isolated using mechanical disruption and enzymatic digestion. Renal cortex tissue was cut into small pieces, washed thoroughly with PBS to remove blood and incubated with collagenase type IV (150 U/ml, Worthington, Lakewood, NJ, USA) and DNase I type IV (50 U/ml) in HBSS + 2% fetal calf serum (FCS) + 0.6% bovine serum albumin (BSA) for 20’ at 37°C. The tissue pieces were washed and processed through a single-cell strainer. Renal biopsy eluates were analyzed directly. Isolates of larger kidney samples underwent density gradient centrifugation and were cryopreserved.

### Virological analyses

Viral DNA was isolated from 200 ul plasma sample by Magnapure96 isolation (Roche applied Science) using the total nucleic acid isolation kit according to the instructions of the manufacturer. Subsequently, isolated DNA was amplified by an internally controlled quantitative realtime TaqMan PCR targeting the Large T-antigen Gene. Quantification was based on standard curves using quantified plasmid DNA containing the target sequence. Values over 1000 copies/ml were considered to be positive.

### Serological analyses

Serum samples were analysed by Luminex for IgG reactivity against the BKPyV-genotype Ib1 major capsid protein 1 (VP1) according to a published protocol [50]. Glutathione—casein (GC) coupled Bio-Plex polystyrene beads (Bio-Rad Laboratories, Hercules, CA, USA) containing a combination of fluorescent dyes were coupled to either GST-BKPyV VP1.tag or GST.tag. For each antigen, 3,000 GC-coupled beads per sample were loaded with crude bacterial lysates containing relevant GST-fusion protein. Samples were preincubated with GST.tag containing bacterial crude lysates (2 mg/mL) in blocking buffer to reduce nonspecific GST binding. The antigen-coated bead mixtures were incubated with serum diluted 1:100. For detection of bound serum antibodies, beads were incubated with goat anti-human total immunoglobulin G—biotin (1:1,000 dilution; Jackson ImmunoResearch Laboratories Inc., West Grove, PA, USA), streptavidin R—phycoerythrin (1:1,000 dilution; Invitrogen), and washed. Beads were analyzed in a Bio-Plex 100 analyzer (Bio-Rad Laboratories). Results are presented as median fluorescent intensity (MFI) units. For each sample, antigen-specific binding was obtained by subtracting the MFI for beads coated with GST alone from those of beads coated with GST VP1. The cut-off value to determine BKPyV-seropositivity was based on sera of healthy children aged 10–15 months old, as described [51].

### Immunofluorescence staining, flowcytometry

For the detection of BKPyV-specific CD8^+^ T cells we utilized combinatorial encoding with six HLA-A02 tetramers loaded with different immunodominant BKPyV peptides. With this technique we generate unique two-colour codes for the parallel detection of three different BKV-specific CD8^+^ T cells populations. As described previously, this technique significantly increases the sensitivity in comparison to single multimer staining and allows for a detection limit as low as 0.002% of total CD8^+^ T cells in large sample sizes (S1B Fig) [52]. To achieve a large enough sample size, we stained up to twelve million PBMC with the tetramers per experiment and determined the presence of BKPyV VP1 and LTAG-specific CD8^+^ T cell populations as well as their expression of various surface and intracellular markers by multichannel flowcytometry (S1C Fig). As advised previously, we used a pre-defined inclusion cut-off value of at least 10 double-positive tetramer events (S1B Fig and S1 Table) [52].

Tetrameric complexes were obtained from Sanquin (Amsterdam, Netherlands) and from the NIH Tetramer Core Facility. Three different and previously tested immunodominant epitopes, shared by the majority of BKPyV strains were selected [10, 15, 16, 26]. This concerned two BKPyV capsid protein VP1 epitopes: BKPyV VP1-derived AITEVECFL (VP1 p44) and BKPyV VP1 LLMWEAVTV (VP1 p108); and one large T antigen protein (LTAg) epitopes: BKPyV LTAg LLLIWFRPV (LTAg p579). These were incorporated in phycoerythrin (PE, Sanquin), allophycocyanin (APC) and Brilliant Violet™ 421-labeled HLA-A02 tetrameric complexes (NIH).

PBMC were washed in phosphate-buffered saline containing 0.01% (wt/vol) NaN3 and 0.5% (wt/vol) bovine serum albumin. Samples were split into aliquots of two million cells. Each aliquot was incubated with a mix of PE-, APC-, and BV421-labeled tetrameric-complexes for two different BKPyV VP1 epitopes and one BKPyV LTag epitope (Sanquin, Amsterdam, Netherlands), followed by incubation with a combination of the following antibodies: CD27 APC-eFluor780 (eBioscience Inc, San Diego, CA, USA), CD8 BrilliantViolet (BV)785, IL-7Rα BV711, CXCR6 PE-Cy7 (BioLegend, San Diego, CA, USA), CD3 V500, CD45RA BV650, CCR7 Brilliant UltraViolet (BUV)395, PD-1 BrilliantBlue515, CD14 PE-CF594, CD19 PE-CF594, CD21 PE-CF594, CD95 BV711 (BD Biosciences, San Jose, CA, USA), CD28 FITC (Sanquin). Dead cells and duplets were excluded from analysis by using Live/Dead fixable staining (Life Technologies Europe BV, Bleiswijk, Netherlands) and height- and width event characteristics, respectively (S1C Fig).

The FOX-P3 staining kit (eBioscience) was used for intracellular stainings with the following antibodies: Eomesodermin PerCP-eFluor710, granzyme K PerCP-eFluor710, T-Bet PE-Cy7 (eBioscience), Ki-67 BUV395 and granzyme B AlexaFluor700 (BD Biosciences). Cells were washed twice, all aliquots of a sample were pooled and up to ten million PBMC per sample were measured on an LSRFortessa flow cytometer and analyzed with FlowJo Version 9.3.3 software. Only live CD19^−^CD4^−^CD20^−^CD8^+^CD3^+^ lymphocytes positive for both differently labelled but otherwise identical tetramers were considered specific for the BKPyV epitope presented in the HLA-A2 tetramer (S1B Fig). CD8^+^ T cell differentiation was determined by surface expression patterns of CD45RA, CCR7, CD28 and CD27. We used a classification that defines the seven largest functionally distinct subsets, involving naïve and stem-cell memory cells (sharing a similar phenotype), central-memory cells (T_CM_), four different effector-memory (T_EM_) subsets and the T_EM_RA subset as described previously [21, 53–55].

Please note that due to limited numbers of available PBMCs per patient we were not always able to do stainings with all the different antibody panels. This affects the data presented on granzyme K, granzyme B, Ki-67, and CD95 expression (which were stained in a separate panel), where we did not have sufficient samples to determine the expression of these markers by BKPyV VP1-specific CD8^+^ T cells in one NR patient, one R^low^ patient and one R^high^ patient at t = pre-peak; three R^low^ patients and three R^high^ patients at t = <6 months post-peak; and two R^low^ patients at t = 2years post-peak. Expression of these markers could also not be measured in BKPyV LTAG-specific CD8^+^ T cells for one R^high^ patient and one BKVN patient at t = peak; nor in one R^low^ patient at t = 2 years post-peak.

### Cytokine production by BK virus-specific T cells

Cytokine release after phorbol 12-myristate 13-acetate (PMA)/ionomycin stimulation was performed as described by Lamoreaux et al.[56]. In short, PBMC were thawed and rested overnight in suspension flasks (Greiner) in RPMI supplemented with 10% FCS, penicillin, and streptomycin (culture medium). Samples were split into aliquots of two million cells. Each aliquot was stimulated with PMA (10 ng/ml) and ionomycin (1 μg/ml) in culture medium in the presence of CD107a FITC (eBioscience); αCD28 (15E8; 2 μg/mL), αCD29 (TS 2/16; 1 μg/mL), brefeldin A (Invitrogen; 10 μg/mL); and GolgiStop (BD Biosciences) in a final volume of 200 μL for 4 hours (PMA at 10 ng/mL/ionomycin at 1 μg/mL) at 37°C and 5% CO2 in untreated, round-bottom, 96-well plates (Corning). Subsequently, cells were incubated with a mix of PE-, APC-, and BV421-labeled tetrameric-complexes for two BKPyV VP1 epitopes and one BKPyV LTag epitope (S1A Fig), followed by incubation with CD14 PE-CF594, CD19 PE-CF594, CD21 PE-CF594, CD3 V500, CD8 BV785, and Live/Dead fixable red cell stain. Cells were then washed twice, fixed, and permeabilized (Cytofix/Cytoperm reagent; BD Biosciences) and subsequently incubated with the following intracellular mAbs: anti-IFNγ BUV 395, anti-TNFα BV650 (BD Biosciences), and anti—IL-2 PerCP-eFluor 710 (eBioscience). Cells were washed twice; all aliquots of a sample were pooled and up to ten million PBMC per sample were measured on an LSRFortessa flow cytometer and analysed with FlowJo Version 9.3.3 software.

### Statistical analysis

Because of the relatively small study group size, non-parametric distribution was assumed. The two-tailed Mann-Whitney test was used to analyse differences between different patient groups. The Kruskal-Wallis test was used to simultaneuously compare all four study groups. To analyse HLA mismatches between different patient groups, we used chi-square testing and to compare all four study groups we used Fisher-Freeman-Halton Exact Testing. Analyses were done with IBM SPSS v24.0. A p-value less than 0.05 was considered statistically significant.

## Supporting Information

S1 Fig (**A**) Schematic overview of the detection of BKPyV virion protein 1 (VP1)- and large T antigen protein (LTAG)-specific CD8^+^ T cells using combinatorial encoding with six different fluorescently-labelled major histocompatibility complex (MHC) class I tetramers loaded with VP1 and LTAG peptides. (**B**) Representative dot plots showing the gating strategy used to define lymphocytes, single cells (exclusion of duplets), CD8-positive and CD3-positive events, four different CD45RA and CD27-defined events, CCR7-negative and positive events, CD28 –negative and positive events, T-bet and/or eomesodermin (Eomes)-positive events, IL-7Rα (CD127)-negative and positive events, CD95-positive events, PD-1-positive events, Ki-67-positive events and granzyme K and/or granzyme B-positive events, respectively. These data were obtained from one representative healthy individual.
(TIF)

S2 Fig Bar graphs showing the detection frequencies of VP1- (open bars) and LTAG-specific (closed bars) CD8^+^ T cells in healthy individuals, in not-reactivating (NR) patients before—and one year after transplantation, and in respectively, the reactivating patients with low (R^low^), high (R^high^) peak viral loads and in patients with BKPyV-induced interstitial nephritis (BKVN) during follow-up. (TIF)

S3 Fig Scatter plot showing the expression frequency of PD-1 (left plot) and CD95 (right plot) by the total CD45^+^CCR7^+^CD28^+^CD27^+^ ‘naïve’ CD8^+^ T cell population and by all the LTAG-specific CD8^+^ T cells with a CD45^+^CCR7^+^CD28^+^CD27^+^ phenotype. (TIF)

S4 Fig Line graphs showing the statistical dispersion of the CD45RA/CCR7/CD28/CD27-defined subset distribution of VP1- and LTAG-specific CD8^+^ T cell populations over time in NR patients, R^low^ patients, R^high^ patients and BKVN patients (mean and standard deviation shown). (TIF)

S5 Fig Pie charts showing the distribution of cytokine combinations produced by VP1-specific CD8^+^ T cells detected after stimulation in vitro in healthy individuals, in NR patients before—and one year after transplantation, and in the R^low^, R^high^ and BKVN RTRs during follow-up (left panel), as well as those produced by LTAG-specific CD8^+^ T cells in the R^low^ patients (right panel). (TIF)

S1 Table Total number of BKPyV-specific CD8^+^ T cell populations detected per subject*. BKPyV = polyomavirus BK. BKVN = BKPyV-induced interstitial nephritis. n/a = not applicable. VL = viral load. c/ml = copies/ml. * Please note that sometimes multiple T cell populations were detected on different time points during the pre-peak, ≤ 6 months post peak, ≥ 6 months post peak ≤ 1 year post peak and ≥ 1 year post peak ≤ 2 years post peak periods for a single patient (also see Materials and Methods: Subjects and Study groups section for a detailed description of the sample inclusion criteria).
(DOCX)
