# Supplementary figures and images for: Clinically Relevant Reactivation of Polyomavirus BK (BKPyV) in HLA-A02-Positive Renal Transplant Recipients Is Associated with Impaired Effector-Memory Differentiation of BKPyV-Specific CD8+ T Cells
Source: PLoS Pathog. 2016 Oct 10;12(10):e1005903. doi: 10.1371/journal.ppat.1005903 (PMC5056763; doi:10.1371/journal.ppat.1005903)

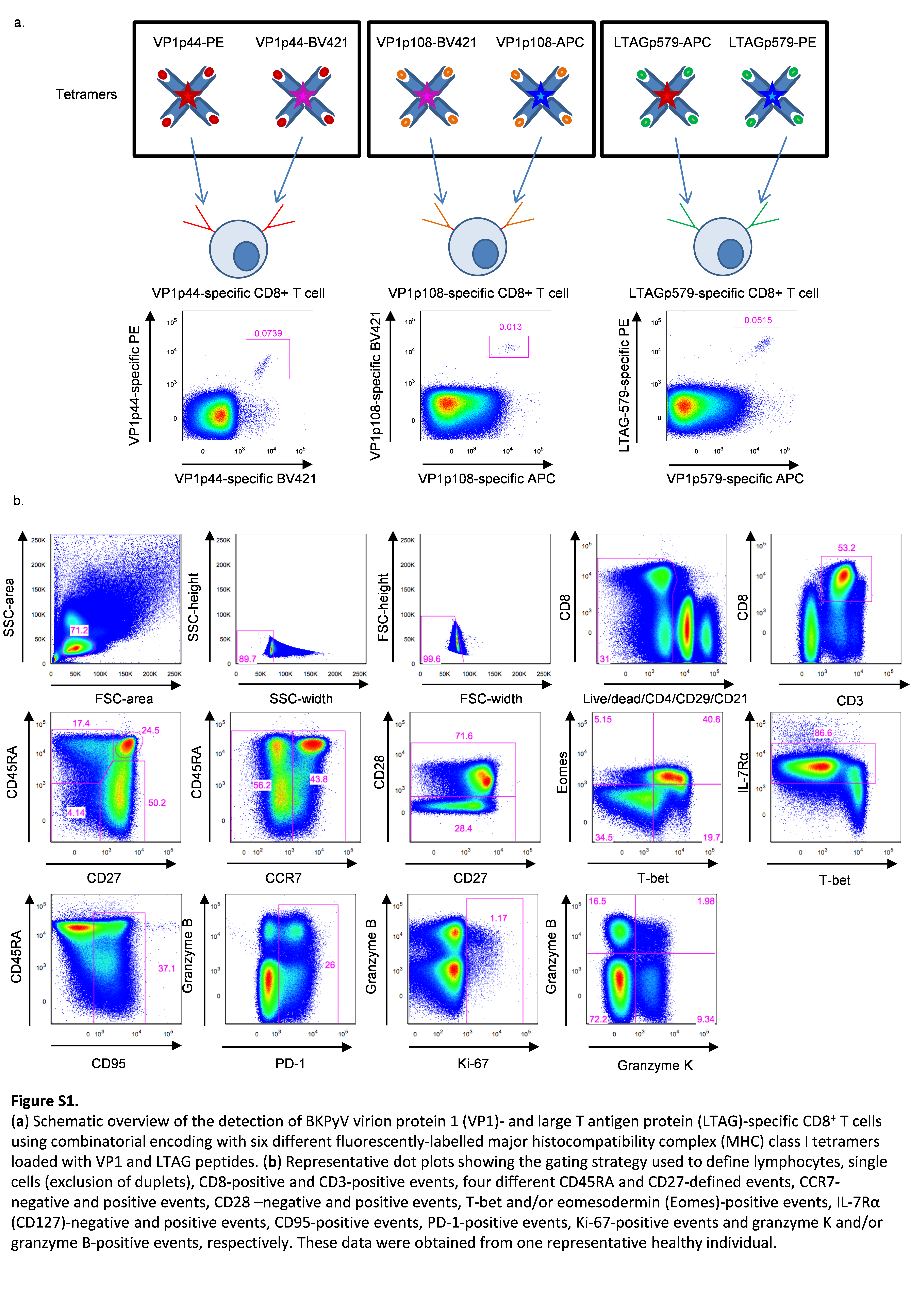

Supplement: S1 Fig — (A) Schematic overview of the detection of BKPyV virion protein 1 (VP1)- and large T antigen protein (LTAG)-specific CD8+ T cells using combinatorial encoding with six different fluorescently-labelled major histocompatibility complex (MHC) class I tetramers loaded with VP1 and LTAG peptides. (B) Representative dot plots showing the gating strategy used to define lymphocytes, single cells (exclusion of duplets), CD8-positive and CD3-positive events, four different CD45RA and CD27-defined events, CCR7-negative and positive events, CD28 –negative and positive events, T-bet and/or eomesodermin (Eomes)-positive events, IL-7Rα (CD127)-negative and positive events, CD95-positive events, PD-1-positive events, Ki-67-positive events and granzyme K and/or granzyme B-positive events, respectively. These data were obtained from one representative healthy individual. (TIF) [file ppat.1005903.s001.tif]

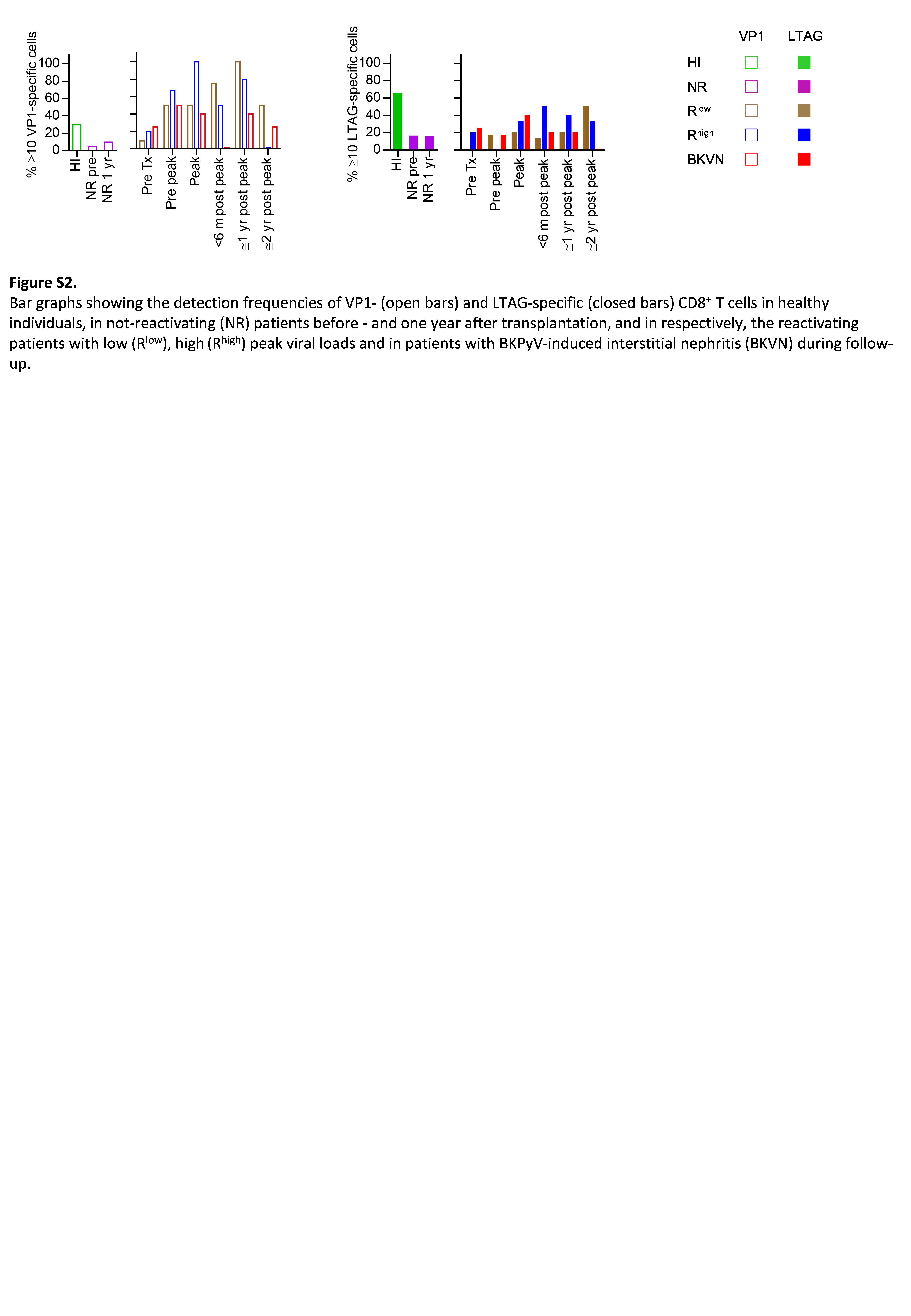

Supplement: S2 Fig — (TIF) [file ppat.1005903.s002.tif]

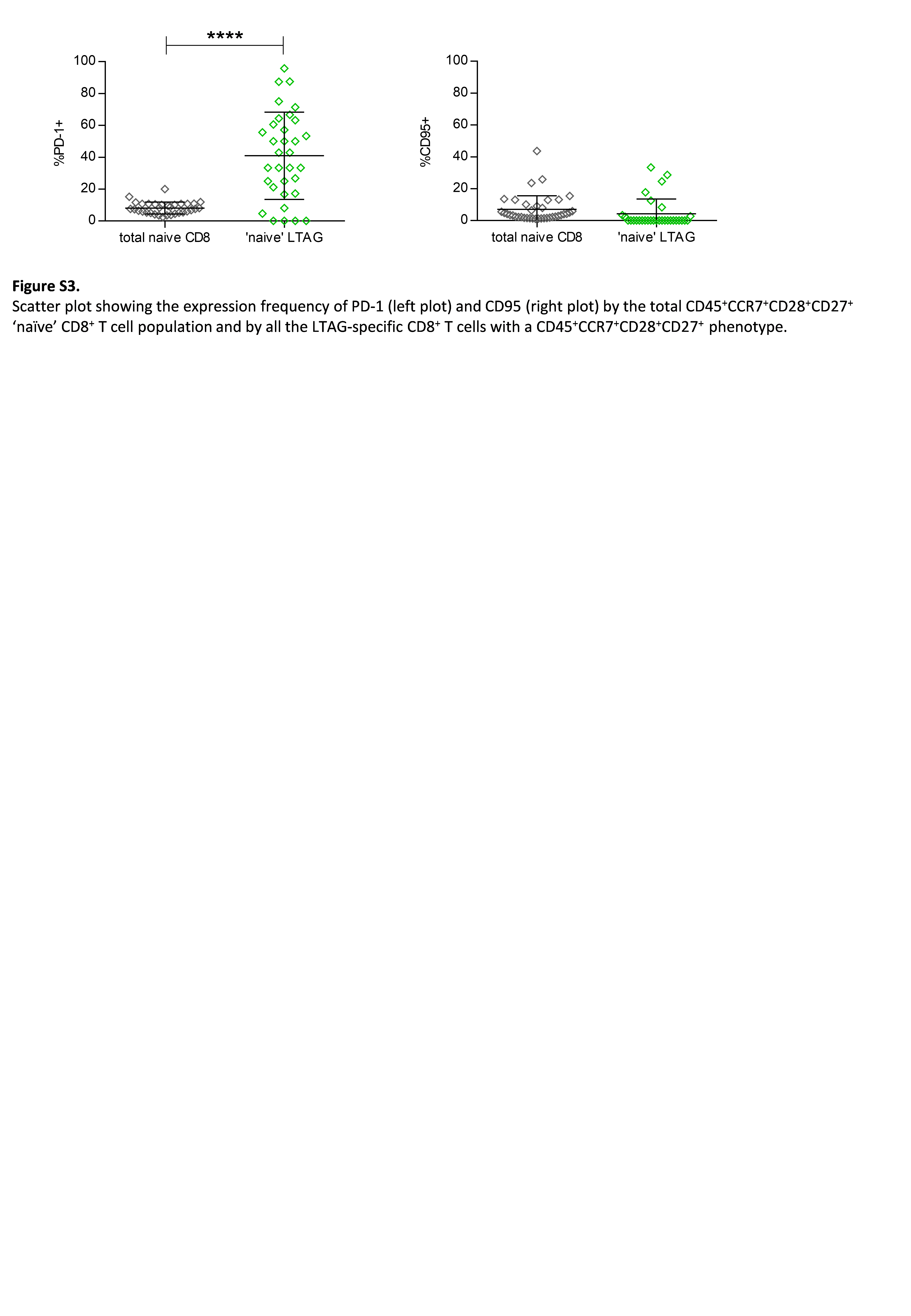

Supplement: S3 Fig — (TIF) [file ppat.1005903.s003.tif]

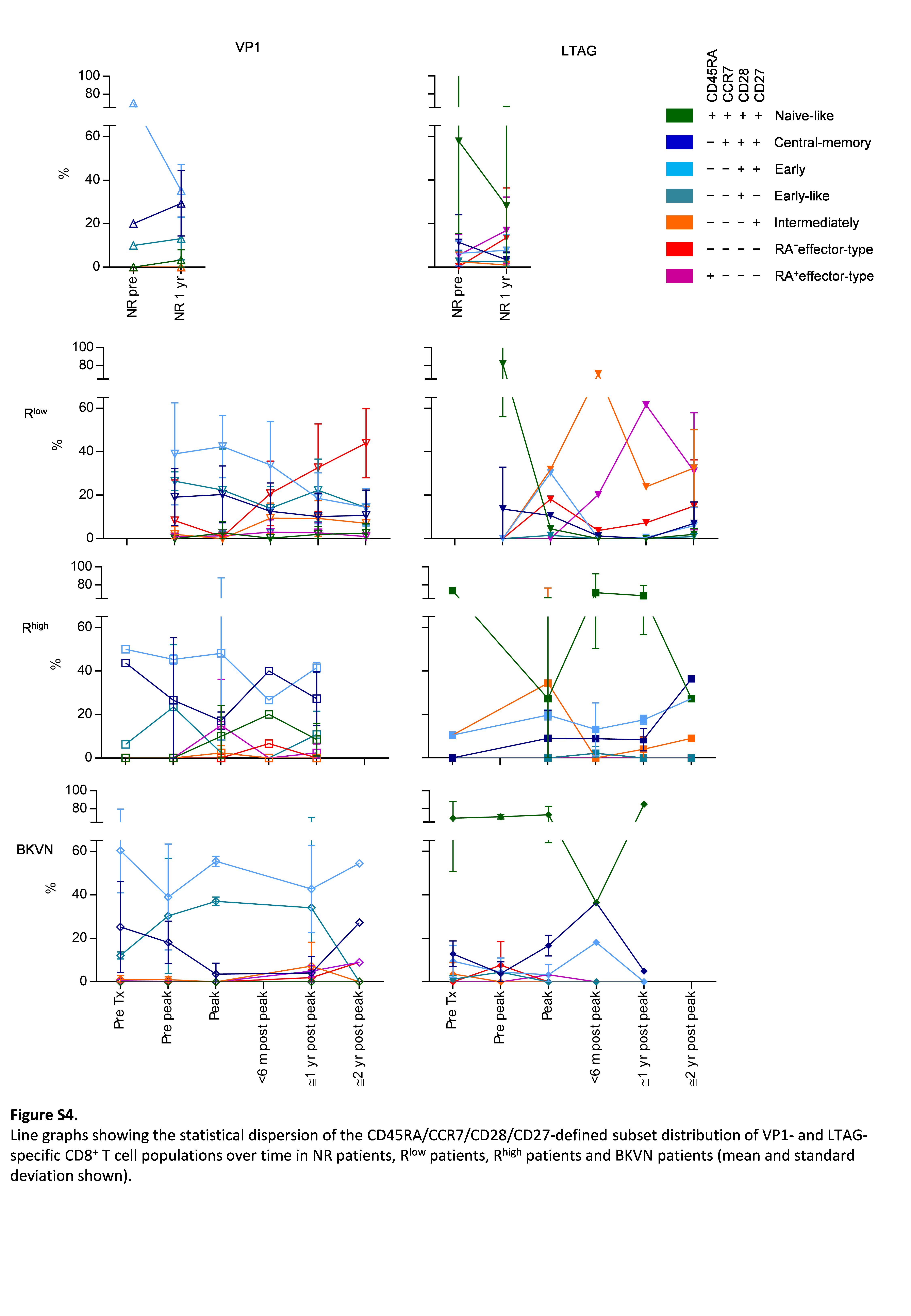

Supplement: S4 Fig — (TIF) [file ppat.1005903.s004.tif]

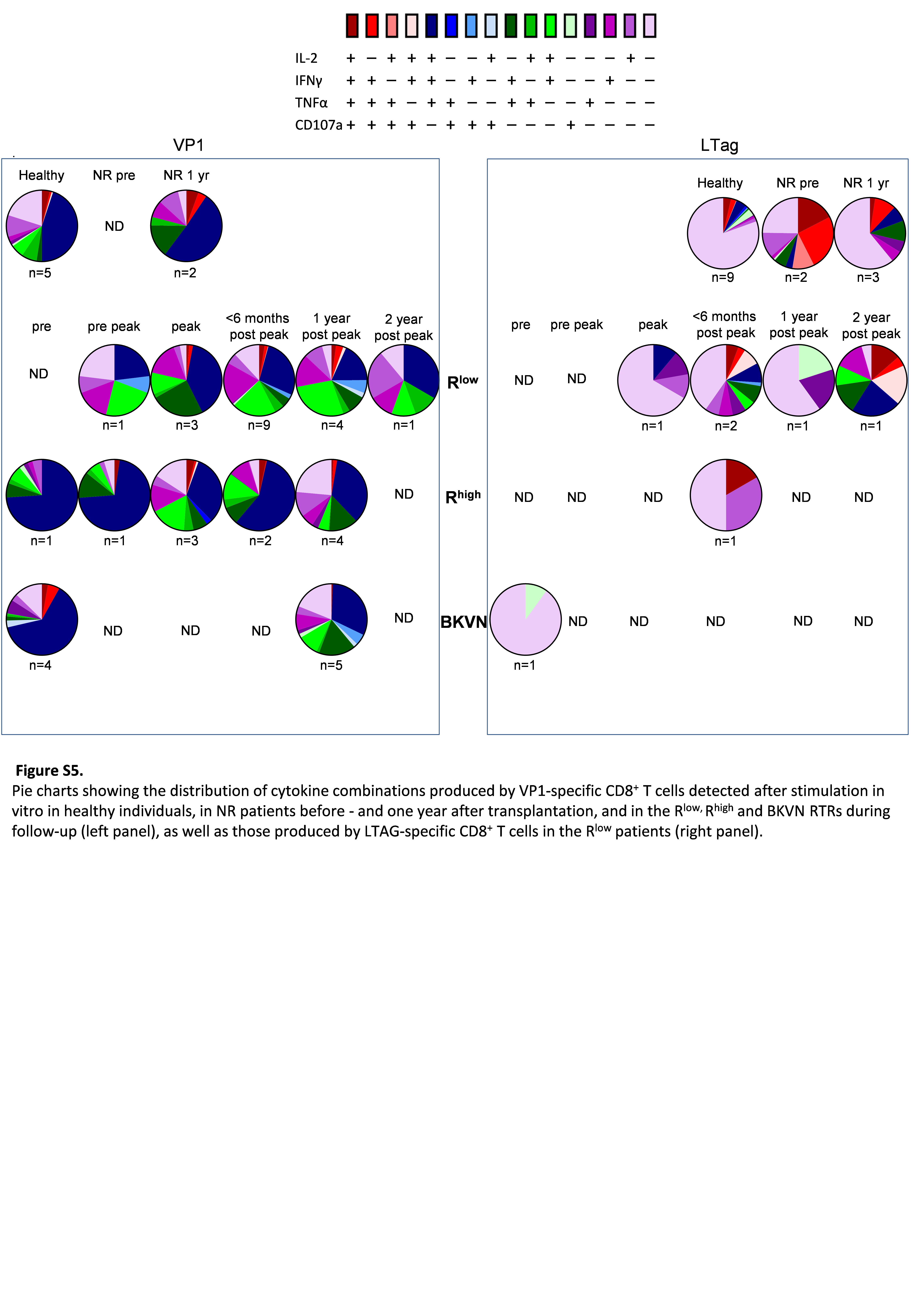

Supplement: S5 Fig — (TIF) [file ppat.1005903.s005.tif]
