# Supplementary material for: Clinically Relevant Reactivation of Polyomavirus BK (BKPyV) in HLA-A02-Positive Renal Transplant Recipients Is Associated with Impaired Effector-Memory Differentiation of BKPyV-Specific CD8+ T Cells
Source: PLoS Pathog. 2016 Oct 10;12(10):e1005903. doi: 10.1371/journal.ppat.1005903 (PMC5056763; doi:10.1371/journal.ppat.1005903)
Supplement: S1 Table — BKPyV = polyomavirus BK. BKVN = BKPyV-induced interstitial nephritis. n/a = not applicable. VL = viral load. c/ml = copies/ml. * Please note that sometimes multiple T cell populations were detected on different time points during the pre-peak, ≤ 6 months post peak, ≥ 6 months post peak ≤ 1 year post peak and ≥ 1 year post peak ≤ 2 years post peak periods for a single patient (also see Materials and Methods: Subjects and Study groups section for a detailed description of the sample inclusion criteria). (DOCX) [file ppat.1005903.s006.docx]

**Supplemental Table I. Total number of BKPyV-specific CD8^+^ T cell populations detected per subject***

|  | **Study groups** | | | | | | | | | | | | | |
| --- | --- | --- | --- | --- | --- | --- | --- | --- | --- | --- | --- | --- | --- | --- |
| **Subject #** | **Healthy inidividuals** | |  | **No BKPyV reactivation**  **[NR]** | |  | **Low peak viral [VL<10e4 c/ml]** | |  | **High peak viral load**  **[VL>10e4 c/ml]** | |  | **BKVN** | |
|  | VP1 | LTAG |  | VP1 | LTAG |  | VP1 | LTAG |  | VP1 | LTAG |  | VP1 | LTAG |
| 1 | 0 | 1 |  | 0 | 0 |  | 0 | 0 |  | 2 | 0 |  | 0 | 0 |
| 2 | 1 | 0 |  | 0 | 0 |  | 7 | 4 |  | 2 | 3 |  | 3 | 0 |
| 3 | 0 | 1 |  | 0 | 0 |  | 6 | 0 |  | 6 | 0 |  | 0 | 1 |
| 4 | 0 | 1 |  | 0 | 0 |  | 0 | 0 |  | 1 | 4 |  | 5 | 0 |
| 5 | 0 | 1 |  | 0 | 2 |  | 4 | 5 |  | 8 | 0 |  | 1 | 0 |
| 6 | 0 | 1 |  | 2 | 0 |  | 3 | 0 |  | 0 | 0 |  | 4 | 1 |
| 7 | 0 | 1 |  | 0 | 0 |  | 6 | 0 |  | n/a | n/a |  | 0 | 4 |
| 8 | 1 | 1 |  | 0 | 0 |  | 0 | 0 |  | n/a | n/a |  | 4 | 2 |
| 9 | 1 | 0 |  | 0 | 1 |  | 4 | 0 |  | n/a | n/a |  | n/a | n/a |
| 10 | 1 | 0 |  | 0 | 0 |  | 4 | 0 |  | n/a | n/a |  | n/a | n/a |
| 11 | 1 | 0 |  | 1 | 0 |  | 1 | 0 |  | n/a | n/a |  | n/a | n/a |
| 12 | 0 | 0 |  | 0 | 0 |  | n/a | n/a |  | n/a | n/a |  | n/a | n/a |
| 13 | 0 | 1 |  | 0 | 0 |  | n/a | n/a |  | n/a | n/a |  | n/a | n/a |
| 14 | 1 | 1 |  | 0 | 1 |  | n/a | n/a |  | n/a | n/a |  | n/a | n/a |
| 15 | 0 | 1 |  | 0 | 0 |  | n/a | n/a |  | n/a | n/a |  | n/a | n/a |
| 16 | 0 | 1 |  | 0 | 0 |  | n/a | n/a |  | n/a | n/a |  | n/a | n/a |
| 17 | 0 | 0 |  | 0 | 0 |  | n/a | n/a |  | n/a | n/a |  | n/a | n/a |
| 18 | 0 | 0 |  | 0 | 0 |  | n/a | n/a |  | n/a | n/a |  | n/a | n/a |
| 19 | 0 | 1 |  | 0 | 0 |  | n/a | n/a |  | n/a | n/a |  | n/a | n/a |
| 20 | 0 | 1 |  | 0 | 0 |  | n/a | n/a |  | n/a | n/a |  | n/a | n/a |
| 21 | n/a | n/a |  | 0 | 2 |  | n/a | n/a |  | n/a | n/a |  | n/a | n/a |

BKPyV = polyomavirus BK. BKVN = BKPyV-induced interstitial nephritis. n/a = not applicable. VL = viral load. c/ml = copies/ml.

* Please note that sometimes multiple T cell populations were detected on different time points during the pre-peak, ≤ 6 months post peak, ≥ 6 months post peak ≤ 1 year post peak and ≥ 1 year post peak ≤ 2 years post peak periods for a single patient (also see Materials and Methods: Subjects and Study groups section for a detailed description of the sample inclusion criteria).
